# Transcriptional profiling identification of inflammatory signaling pathways in ulcerative colitis

**DOI:** 10.1371/journal.pone.0330332

**Published:** 2025-09-02

**Authors:** Salvia Misaghian, M. Saleet Jafri

**Affiliations:** 1 School of Systems Biology, George Mason University, Fairfax, Virginia, United States of America; 2 Center for Biomedical Engineering and Technology, University of Maryland School of Medicine, Baltimore, Maryland, United States of America; Versiti Blood Research Institute, UNITED STATES OF AMERICA

## Abstract

Ulcerative colitis (UC) is a chronic type of inflammatory bowel disease (IBD). This study identified core genes and pathways involved in UC by performing transcriptional profiling of colon biopsies from UC patients and healthy controls using data from the Gene Expression Omnibus (GEO) database. A total of 202 samples, including 129 UC patients and 73 healthy controls, were analyzed, measuring the expression of 40,991 genes using a 44K formatted microarray. Differential gene expression (DGE) analysis and gene set enrichment analysis (GSEA) identified several biomarkers potentially involved in UC development. PLCB3 was significantly downregulated, which suggested its role in maintaining intestinal homeostasis. In contrast, DUOX2 was upregulated, which indicated its involvement in the inflammatory response and oxidative stress. Pathway analysis revealed that PLCB3 is associated with lipid metabolism, NOD-like receptor signaling, and NF-κB signaling pathways, while DUOX2 is linked to reactive oxygen species production and chemokine signaling. The interplay between PLCB3 and DUOX2 suggests their combined impact on inflammatory processes in UC. These insights into the molecular mechanisms underlying UC identify key genes and pathways that could serve as potential targets for diagnostic and therapeutic interventions.

## Introduction

Inflammatory bowel disease (IBD) can be subdivided into several different subcategories. Ulcerative colitis (UC) and Crohn’s disease (CD) are two chronic types of IBD [1]. However, although each of these conditions share many similar symptoms such as abdominal pain, diarrhea, and fatigue, each of them shows their own unique features [2]. The highest reported annual incidence rates of UC are in Europe (24.3 per 100,000) and North America (19.2 per 100,000). In contrast, the rates are much lower in Asia and the Middle East (6.3 per 100,000), likely due to differences in levels of industrialization [3]. UC follows a bimodal pattern of incidence, with most diagnoses occurring between the ages of 15 and 30, and a smaller peak between the ages of 50 and 70 [4]. The exact cause of this condition is still unknown, but it appears to be a combination of different factors such as environmental and genetic factors. Because of the difficulties that exist in UC treatment and lack of a cure for this disease, the rate of relapse and risk of cancer are high [5]. The World Health Organization classified this condition as a refractory illness. The prevalence rate of UC is ~ 247 people from every 100,000 people [6]. It is important to note that UC and CD are chronic and relapsing conditions, requiring long-term management and monitoring.

Currently, the only measurement of the stage of inflammation in UC patients has been limited to nonspecific factors such as C-reactive protein (CRP) and the erythrocyte sedimentation rate (ESR). These factors do not have acceptable specificity to distinguish between different types of IBD such as Crohn’s disease and Ulcerative colitis [7]. UC is mainly recognized by finding inflammation in the rectal area as continuous lesions, but CD is known to be presented as discontinuous inflammation and ulcer that usually involves the ileocecal area [8]. Serological markers are often used to differentiate between UC and CD. Anti-*Saccharomyces cere*visiae antibodies (ASCA) is a marker that can be found in almost 50% of the patients who suffer from CD, but not UC. In patients with UC, it is more common to detect perinuclear antineutrophil cytoplasmic antibodies (pANCA). The presence of pANCA can be identified in UC patients with more sensitivity up to 55.3%. Nevertheless, such a low sensitivity for this marker is not always reliable to differentiate between CD and UC [8]. Although the causes of both diseases are still uncertain, researchers have suggested that genetic factors are involved in their pathogenesis [5]. However, there are similarities between both diseases, but each of them requires distinct treatment. Misdiagnosis of these diseases can cause wrong treatment and consecutive suffering. In this study, by using bioinformatics analysis on data from Gene Expression Omnibus (GEO) databases that were revealed by microarray technology, we verify and select core genes that are involved in UC by performing Pathway Analysis and Comparative Analysis of Gene Expression.

Inflammation is an important immune-mediated mechanism to protect the body from pathogens and physical harm. Immune responses are not always favorable for the body, and they could cause tissue or organ transplant rejection, hypersensitivity responses, and septic shock. Improper functioning of the immune system may cause chronic inflammation, and lead to diseases such as autoimmune disorders and gastrointestinal disorders. Studies show that different alleles of genes and missense heritable mutations that are related to inflammatory pathways can cause and increase the severity of inflammatory diseases [6]. Complex inflammation responses require the involvement of many genes. The variation and different expression levels in these genes can provide alternative responses and functions. Systematic assessment of these genes that are associated with inflammation responses can provide better ideas for disease susceptibility [6].

In one study by Sarlos et al., they reviewed and conducted an electronic search on the Pubmed Database, focusing on publications from the past decade. The investigation utilized medical subject heading terms, including UC, ulcerative colitis, inflammation, genes, polymorphisms, and susceptibility. Sarlos et al. indicated the influence of genetic variations in various interleukins (ILs) on ulcerative colitis and examined the role of IL-1, IL-2, IL-6, IL-8, IL-10, IL-12, IL-17, IL-18, IL-23, and IL-26 in UC susceptibility and phenotype. Due to their study, IL-1B and IL-1RN, involved in inflammation regulation, were analyzed in Mexican Mestizo UC patients, revealing associations between specific genotypes and UC susceptibility. Their review indicated that polymorphisms in the IL-2/IL-21 region show associations with inflammatory bowel disease, particularly UC, in different populations. Genetic variations in IL-6, IL-8, and IL-10 genes were linked to UC susceptibility in various populations. Polymorphisms in IL-12, IL-17, IL-18, and IL-23 genes are also associated with UC susceptibility or phenotype in different people. Additionally, specific markers in IL-26 were associated with UC. Sarlos et al. demonstrated that the results from various studies examining these genetic variations in UC needed to be more consistent, which indicate these genetic factors’ complexity and population-specific nature. Sarlos et al. also discussed the involvement of other genes, including CTLA4, JAK2, STAT3, TNFα, OCTN1, OCTN2, MDR1, NOD1/CARD4, toll-like receptors (TLRs), and cell adhesion molecules (CAMs) in UC. These genes play diverse roles in immune responses, cytokine signaling, transporter activity, and innate immunity. Some of these genes showed associations with UC susceptibility, disease severity, or clinical characteristics in specific populations. Further research is needed to fully understand the implications and mechanisms of these genetic variations in UC and their relevance to different people [4].

Previous research into gene expression in UC and CD has shown the complexity of these chronic inflammatory bowel diseases. However, these conditions share similar symptoms but have different traits. Existing studies have determined serological markers and genetic factors associated with UC and CD. Yet, misdiagnosis remains a challenge for both healthcare providers and patients. In the current study, we build on this foundation by using bioinformatics analysis to identify core genes involved in UC. We will identify the novel biomarkers potentially involved in UC development by finding the DEGs. Then, by using pathway analysis, we will indicate the connection between different pathways and the functional mechanisms of UC.

## Materials and methods

### Data acquisition

Gene expression data for ulcerative colitis (UC) and normal samples were obtained from GEO (http://www.ncbi.nim.nih.gov/geo/) database with accession number GSE11223 (biopsies from sigmoid colon, descending colon, ascending colon, terminal ileum. 202 samples including 129 UC patients (with different categories of active or inactive UC) and 73 normal candidates were used for the analysis (Table 1). The dataset included both series and platform data, which were loaded using the GEOquery R package (version 2.66.0).

**Table 1:** List of the datasets and numbers of samples control vs diseased.

Accession number	Number of samples	Controls	Diseased	Targeted genes	Platform	Samples
GSE11223	202	73	129	44K	Microarray	Colon epithelial biopsies

### Data processing

The raw expression data were processed to ensure consistency in column names and to handle missing values. The expression values were log2 transformed, if necessary, based on the quantile values of the dataset. Samples were assigned to their respective groups based on their condition (UC vs. Normal, and further divided into UC inflamed, UC uninflamed, and Normal). A design matrix was created to facilitate differential expression analysis.

### Differential expression analysis

Differential expression analysis was performed by using the limma package (version 3.54.0) in R (version 4.2.2). The analysis involved fitting a linear model to the expression data and setting up contrasts to compare different conditions. The empirical Bayes method was used to compute statistics and generate a table of top significant genes. Results were adjusted for multiple testing using the Benjamini-Hochberg method, and significant genes were identified based on adjusted p-values and log2 fold changes (log2FC). The top significant genes were saved to CSV files for further analysis.

### Data analysis with inflammation consideration

To further investigate the role of inflammation, samples were categorized into three groups: UC inflamed, UC uninflamed, and Normal. Differential expression analysis was repeated with these additional groupings, and contrasts were set up to compare each pair of conditions. Significant genes were identified and saved to CSV files.

### Correlation analysis

Correlation analysis was conducted to explore the relationships between gene expression levels. Pearson correlation coefficients were calculated for pairs of genes across all samples. The correlation analysis involved converting the dataset to tibble format and selecting the columns that included the expression level of selected genes. After removing rows with missing values, a log transformation was applied to normalize the data. Rows with non-finite values were then filtered out to ensure a final dataset suitable for analysis. A preliminary check confirmed sufficient observations, followed by calculating Pearson correlation coefficients to produce a correlation matrix. This matrix was exported to a CSV file for further analysis and ensuring a reliable assessment of variable relationships.

### Pathway analysis

Pathway analysis was performed to identify biological pathways significantly enriched in the differentially expressed genes. The clusterProfiler package (version 4.2.2) in R was used to perform Gene Ontology (GO) enrichment analysis and Kyoto Encyclopedia of Genes and Genomes (KEGG) pathway analysis. Significant pathways were identified based on adjusted p-values, and visualizations were created to illustrate the enriched pathways.

### Data visualization

Gene expression data were visualized using the ggplot2 package (version 3.3.5) in R. Analytes of interest were log-transformed, and jitter plots were created to show the distribution of expression values across different groups. Pairwise comparisons were performed using t-tests, and p-values were adjusted using the Bonferroni method. Volcano plots were generated to highlight significant genes, with fold changes and p-values visualized on the x and y axes, respectively.

### Statistical analysis

Pairwise comparisons between different conditions were performed using t-tests, and p-values were adjusted using the Benjamini-Hochberg method. Genes with adjusted p-values less than 0.05 and absolute log2 fold changes greater than 1 were considered significant. Statistical analyses were conducted using base R functions and additional packages such as dplyr and tidyr.

### Software and tools

All analyses were performed using R (version 4.2.2) and the following R packages: Biobase (version 2.58.0), GEOquery (version 2.66.0), limma (version 3.54.0), umap, ggplot2, dplyr, tidyr, pheatmap, and ggpubr. Data visualization and statistical analyses were conducted using standard functions and custom scripts in R.

### Data availability

The dataset used in this study was obtained from the **Gene Expression Omnibus (GEO)** database. The accession number is **GSE11223,** and it can be accessed directly via the following link:

https://www.ncbi.nlm.nih.gov/geo/query/acc.cgi?acc=GSE11223

This dataset includes biopsies from the sigmoid colon, descending colon, ascending colon, and terminal ileum. In total, **202 samples** were analyzed, comprising **129 UC patients** (with varying categories of active or inactive UC) and **73 normal controls**, as detailed in Table 2 of the manuscript.

**Table 2:** List of the top significantly differentially expressed genes between the normal group and UC group. The table includes gene symbols, gene titles, adjusted p-values, and raw p-values.

ID	Gene.symbol	Gene.title	adj.p-value (× 10)	P = value (× 10)
19489	CCNO	cyclin O	2.89 × 10^−5^	9.32 × 10^−9^
10494	DUOX2	dual oxidase 2	2.54 × 10^−4^	3.20 × 10^−7^
8497	MMP7	matrix metallopeptidase 7	6.52 × 10^−4^	2.29 × 10^−6^
16982	CCL11	C-C motif chemokine ligand 11	4.25 × 10^−5^	1.79 × 10^−8^
11488	BACE2	beta-site APP-cleaving enzyme 2	1.21 × 10^−4^	7.30 × 10^−8^
21986	C1orf54	chromosome 1 open reading frame 54	2.89 × 10^−5^	9.09 × 10^−9^
12037	MIR4435−2HG	MIR4435−2 host gene	2.29 × 10^−6^	2.22 × 10^−10^
22027	ZFAS1	ZNFX1 antisense RNA 1	8.19 × 10^−7^	5.29 × 10^−11^
18792	CARD8	caspase recruitment domain family member 8	7.49 × 10^−4^	3.03 × 10^−6^
26813	CNTNAP2	contactin associated protein-like 2	9.75 × 10^−5^	5.35 × 10^−8^
36434	ACO2	aconitase 2	3.94 × 10^−5^	1.51 × 10^−8^
28123	PLCB3	phospholipase C beta 3	8.19 × 10^−7^	3.23 × 10^−11^
16694	DPP10-AS1	DPP10 antisense RNA 1	3.94 × 10^−5^	1.53 × 10^−8^

### Summary of analysis

In summary, this study involved the acquisition of gene expression data for UC and normal samples, preprocessing of the data, differential expression analysis considering both overall and inflammation-specific groupings, correlation analysis, pathway analysis, and visualization of the results using various R packages. The findings provide insights into the molecular differences between UC and normal samples, as well as the impact of inflammation on gene expression.

## Results

### Differential expression analysis

#### Differential expression results without inflammation consideration.

**Fig 1:**
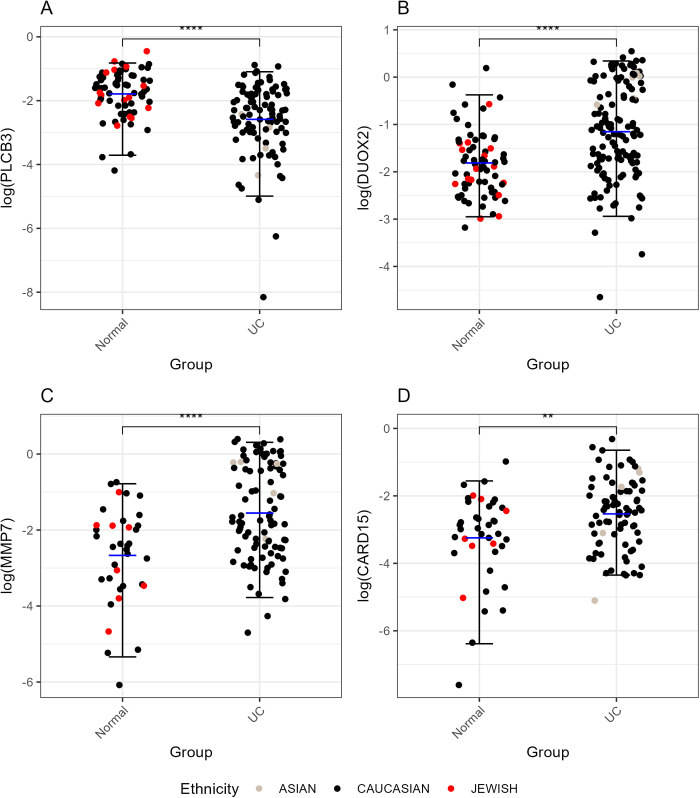
Gene expression comparison across disease groups. This figure presents four panels (A, B, C, D) showing the log-transformed expression levels of different analytes across disease groups. Panel A displays PLCB3, Panel B shows DUOX2, Panel C features MMP7, and Panel D illustrates CARD15. Each panel uses jitter plots to represent individual data points, with median error bars (black), which represent the median and interquartile range (IQR) of the data within each disease group, and mean error bars (blue), which Represent the mean values within each subgroup of a disease group. Points are colored by ethnicity, and significant differences between groups are marked by p-values from t-tests, adjusted using the Bonferroni method.

**Fig 2:**
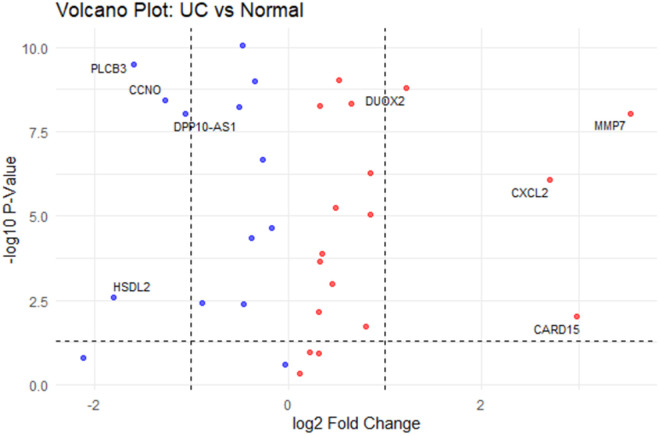
Gene expression differences. Volcano plots visualize significantly differentially expressed genes in GSE11223 without inflammation consideration. The cutoff p-value is set at 0.05, and the log2 fold change is set on values greater than 1 and less than −1. The red color indicates the upregulation, and blue color indicates of downregulation.

After performing the empirical Bayes method and adjusting the result by multiple testing using the Benjamini-Hochberg method, we generated a table of top significant genes without considering the inflammation status among the samples. This analysis dataset contains information on various genes with their respective adjusted p-values, p-values, gene symbols, and gene titles. Here is a list of some of the key observations from the top significantly differentially expressed genes:

After this analysis, we selected some of the top significant genes from the GSE11223 dataset and saved them in a CSV file. We analyzed the differential gene expression data to identify genes with significant changes in expression levels. The key findings from the gene expression analysis are summarized in Table 2. Notably, PLCB3 showed significant downregulation with a log2 fold change of −1.59, which indicates its potential involvement in UC (Table 3). Additionally, jitter plots for multiple genes including PLCB3, DUOX2, MMP7, and CARD15 (NOD2) (Fig 1) and a volcano plot (Fig 2) were generated to visualize this result. In Fig 1, individual data points for each of the genes are shown to provide a clearer view of the data distribution within each disease group. The median error bars (black) represent the median and interquartile range (IQR) of the log-transformed values within each disease group. The median is shown as the central value, while the IQR indicates the range between the first and third quartiles. In addition, the mean error bars (blue) Indicate the mean values within each disease group, with error bars showing the standard error of the mean. Also, a statistical comparison between the UC and Normal groups is performed by using a t-test with Bonferroni adjustment. Significant differences are indicated with p-value labels.

**Table 3:** List of the genes that were differentially expressed between normal group and UC group without inflammation consideration. Genes with Log2FC greater than 1 are considered upregulated, and Genes with Log2FC less than −1 are considered downregulated.

Gene	log2FC	p-value	adj.p-value	Regulation
HSDL2	−1.8	2.65 × 10^−3^	3.92 × 10^−3^	Down
PLCB3	−1.59	3.18 × 10^−10^	5.88 × 10^−9^	Down
CCNO	−1.27	3.74 × 10^−9^	2.31 × 10^−8^	Down
DPP10-AS1	−1.06	9.55 × 10^−9^	3.21 × 10^−8^	Down
DUOX2	1.22	1.59 × 10^−9^	1.17 × 10^−8^	Up
CXCL2	2.7	8.69 × 10^−7^	1.89 × 10^−6^	Up
CARD15	2.99	9.23 × 10^−3^	1.18 × 10^−2^	Up
MMP7	3.54	9.02 × 10^−9^	3.21 × 10^−8^	Up

#### Differential expression with inflammation consideration.

To facilitate further analysis, we incorporated inflammation as a factor in our study. This approach enabled us to identify genes that are significantly altered across various conditions: Normal vs. UC uninflamed, Normal vs. UC inflamed, and UC uninflamed vs. UC inflamed. We conducted a differential gene expression analysis to pinpoint these significant changes. The results include differentially expressed genes related to inflammation, with additional columns such as the F-statistic, adjusted p-values, p-values, log fold changes, gene symbols, and gene titles. These findings are summarized in Tables 4 and 5.

**Table 4:** Significant Genes in Differential Expression Analysis. This table lists genes with the most significant differential expression across different conditions, including their ID, adjusted p-value, raw p-value, fold change (F), gene symbol, and gene title.

ID	adj.p-value	p-value	F	Gene.symbol	Gene.title
10494	2.57 × 10^−17^	8.30 × 10^−22^	44	DUOX2	dual oxidase 2
16694	1.90 × 10^−14^	1.84 × 10^−18^	36	DPP10-AS1	DPP10 antisense RNA 1
11488	2.29 × 10^−14^	2.95 × 10^−18^	35	BACE2	beta-site APP-cleaving enzyme 2
12587	4.52 × 10^−14^	7.30 × 10^−18^	34	PI3	peptidase inhibitor 3
14865	2.28 × 10^−13^	4.41 × 10^−17^	32	ZC3H12A	zinc finger CCCH-type containing 12A
18191	6.72 × 10^−13^	1.52 × 10^−16^	31	PDZK1IP1	PDZK1 interacting protein 1
18979	1.30 × 10^−12^	3.52 × 10^−16^	30	LCN2	lipocalin 2
2627	1.30 × 10^−12^	3.79 × 10^−16^	30	LINC00963	long intergenic non-protein coding RNA 963
8497	1.67 × 10^−12^	5.40 × 10^−16^	30	MMP7	matrix metallopeptidase 7
35939	1.99 × 10^−12^	7.07 × 10^−16^	30	CXCL2	C-X-C motif chemokine ligand 2
42513	7.28 × 10^−12^	3.29 × 10^−15^	28	BACE2	beta-site APP-cleaving enzyme 2
39686	7.89 × 10^−12^	3.82 × 10^−15^	28	CXCL1	C-X-C motif chemokine ligand 1
28123	3.30 × 10^−8^	1.42 × 10^−10^	18	PLCB3	phospholipase C beta 3

**Table 5:** Differential Expression of Selected Genes. This table shows the log2 fold change (log2FC), p-value, and adjusted p-value for selected genes across three comparisons: Normal vs. UC uninflamed, Normal vs. UC inflamed, and UC uninflamed vs. UC inflamed.

	Normal vs UC uninflamed			Normal vs UC inflamed			UC uninflamed vs UC inflamed		
Gene	log2FC	P.Value	adj.P.Value	log2FC	P.Value	adj.P.Value	log2FC	P.Value	adj.P.Value
**MMP7**	1.54	1.26 × 10^−1^	2.74 × 10^−1^	4.37	6.16 × 10^−10^	1.42 × 10^−9^	2.83	1.87 × 10^−8^	6.28 × 10^−8^
**CXCL2**	0.22	8.61 × 10^−1^	9.10 × 10^−1^	3.6	2.45 × 10^−10^	7.56 × 10^−10^	3.38	1.05 × 10^−9^	4.87 × 10^−9^
**CARD15**	2.09	2.10 × 10^−1^	4.31 × 10^−1^	3.56	7.34 × 10^−3^	8.48 × 10^−3^	1.47	6.52 × 10^−2^	9.28 × 10 ⁻ ²
**DUOX2**	0.22	3.37 × 10^−1^	5.57 × 10^−1^	1.82	1.43 × 10^−13^	1.77 × 10^−12^	1.61	4.15 × 10^−12^	5.12 × 10^−11^
**PI3**	0.01	9.73 × 10^−1^	9.73 × 10^−1^	1.4	2.67 × 10^−10^	7.61 × 10^−10^	1.39	5.56 × 10^−10^	2.94 × 10^−9^
**IL1B**	0.17	4.24 × 10^−1^	6.27 × 10^−1^	1.33	8.02 × 10^−8^	1.41 × 10^−7^	1.16	1.23 × 10^−6^	3.26 × 10^−6^
**NCF1**	0.45	2.94 × 10^−1^	5.18 × 10^−1^	1.1	4.65 × 10^−3^	6.37 × 10^−3^	0.65	5.52 × 10^−2^	8.34 × 10^−2^
**CD300A**	−0.77	3.05 × 10^−2^	9.26 × 10^−2^	−1.02	7.26 × 10^−3^	8.48 × 10^−3^	−0.25	6.49 × 10^−1^	6.67 × 10^−1^
**PLCB3**	−1.33	2.53 × 10^−7^	9.37 × 10^−6^	−1.93	1.99 × 10^−9^	4.33 × 10^−9^	−0.6	2.24 × 10^−1^	2.59 × 10^−1^
**CCNO**	−0.69	1.15 × 10^−3^	6.91 × 10^−3^	−2.31	5.00 × 10^−12^	2.64 × 10^−11^	−1.62	5.89 × 10^−4^	1.28 × 10^−3^
**DPP10-AS1**	−0.27	7.48 × 10^−2^	1.94 × 10^−1^	−3.15	1.47 × 10^−17^	5.43 × 10^−16^	−2.88	1.34 × 10^−12^	4.97 × 10^−11^
**NALP12**	−0.34	6.06 × 10^−1^	7.98 × 10^−1^	−4.32	5.58 × 10^−2^	6.07 × 10^−2^	−3.98	1.45 × 10^−1^	1.79 × 10^−1^

**Fig 3:**
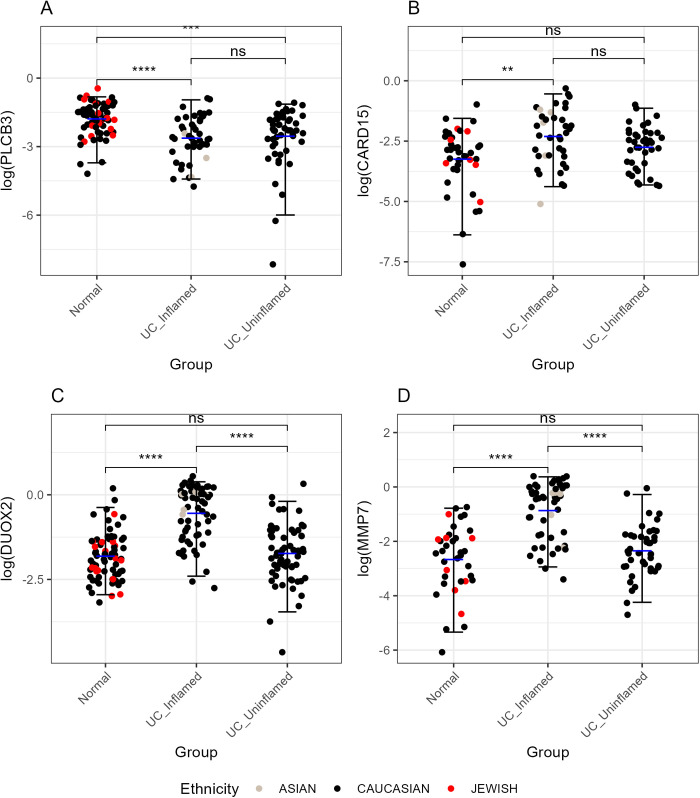
Gene expression comparison across different matrices. This figure presents four panels (A, B, C, D) showing the log-transformed expression levels of different genes across various sample matrices. Panel A displays PLCB3, Panel B shows CARD15, Panel C features DUOX2, and Panel D illustrates MMP7. Each panel uses jitter plots to represent individual data points, with median error bars (black), which represent the median and interquartile range (IQR) of the data within each disease group, and mean error bars (blue), which Represent the mean values within each subgroup of a disease group. Points are colored by ethnicity, and significant differences between groups are marked by p-values from t-tests, adjusted using the Bonferroni method.

Table 4 presents the genes with the most significant differential expression across the conditions. The dual oxidase 2 (DUOX2) gene showed the highest level of significance with an adjusted p-value of 2.57 × 10^−17^ and a p-value of 8.30 × 10^−22^, with a fold change (F) value of 44. Other highly significant genes included DPP10 antisense RNA 1 (DPP10-AS1), beta-site APP-cleaving enzyme 2 (BACE2), peptidase inhibitor 3 (PI3), and zinc finger CCCH-type containing 12A (ZC3H12A). Some of these genes including PLCB3, DUOX2, MMP7, and CARD15 were visualized by generating jitter plots with mean and median error bars (Fig 3).

In addition, an ANOVA test was conducted to assess the effects of different conditions (Normal, UC Inflamed, UC Uninflamed) and ethnicities on the log-transformed values of the genes PLCB3, DUOX2, MMP7, and CARD15. The results showed that different conditions had a significant impact on the log-transformed values for all four genes: PLCB3 (p = 7.18E-06), DUOX2 (p = 2.81E-19), MMP7 (p = 1.36E-12), and CARD15 (p = 2.03E-03). In contrast, ethnicity did not have a significant effect on any of the genes, with p-values of 0.368, 0.489, 0.978, and 0.874 for PLCB3, DUOX2, MMP7, and CARD15, respectively. This indicates that while the different disease groups significantly influence gene expression levels, the differences in ethnicity do not.

**Fig 4:**
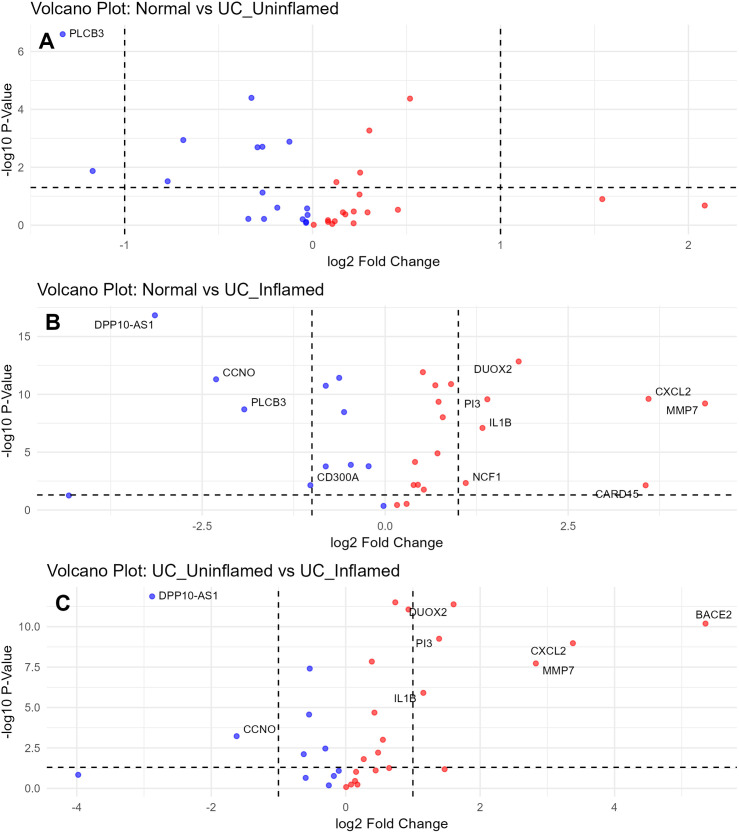
Differential gene expression analysis in normal, UC uninflamed, and UC inflamed conditions. This figure displays three volcano plots (A, B, C) illustrating the differential gene expression in GSE11223 with inflammation consideration. The cutoff p-value is set at 0.05, and the log2 fold change threshold is set to values greater than 1 or less than −1. Red indicates upregulation, while blue indicates downregulation. Plot A compares Normal vs. UC Uninflamed, Plot B compares Normal vs. UC_Inflamed, and Plot C compares UC Uninflamed vs. UC_Inflamed. Dashed lines indicate the significance thresholds. These plots provide an overview of gene expression changes associated with each condition.

Table 5 highlights the log2 fold change (log2FC), p-values, and adjusted p-values for selected genes across the three comparisons. In this analysis, DUOX2 and CXCL2 showed significant upregulation in UC inflamed samples compared to both normal and UC uninflamed samples, which could suggest their role in the inflammatory response [9,10]. MMP7 was significantly upregulated in UC inflamed samples, indicating its involvement in tissue remodeling and inflammation [11]. PLCB3 showed significant downregulation in UC inflamed samples compared to normal samples, highlighting its potential role in maintaining intestinal homeostasis [12]. In addition, volcano plots were generated to visually represent these differential expression patterns (Fig 4).

### Correlation analysis

In our study, we conducted a correlation analysis to investigate the relationships between gene expression levels across different conditions. The analysis identified several significant strong correlations (above 0.7 and below −0.7) that provide insights into the potential interactions and regulatory mechanisms underlying UC.

In our analysis, we identified several genes that exhibit strong correlations with DUOX2, providing insights into potential co-regulatory mechanisms and functional relationships in the context of UC. DUOX2 shows a very strong positive correlation with LCN2 (r = 0.86). Lipocalin 2 is involved in the innate immune response and is known for its role in sequestering iron, which limits bacterial growth [13]. The strong correlation suggests that DUOX2 and LCN2 may be co-regulated during the inflammatory response, contributing to host defense mechanisms in UC. There is also a strong positive correlation between DUOX2 and MMP7 (r = 0.82). MMP7 is involved in the degradation of extracellular matrix components, which is crucial during tissue remodeling and inflammation [14]. This correlation indicates that DUOX2 may work in conjunction with MMP7 to facilitate tissue repair and remodeling processes in the inflamed intestinal mucosa of UC patients. DUOX2 shows a strong positive correlation with PDZK1IP1 (r = 0.82). The cargo protein MAP17 (PDZK1IP1) regulates the immune microenvironment and is linked to chronic inflammation, a key factor in cancer development. Overexpression of MAP17 triggers local inflammation and correlates with inflammatory markers like HLAs, BBS10, HERC2, ADNP, and PYCARD. MAP17 also directly regulates NFAT2 and IL-6 activation, inducing monocyte differentiation into dendritic cells [15]. This correlation suggests potential co-regulation between DUOX2 and PDZK1IP1, which could influence cellular responses to inflammation. In addition, a strong positive correlation exists between DUOX2 and PI3 (r = 0.80). PI3 is an inhibitor of neutrophil elastase, a protease involved in inflammation [16]. This relationship indicates that DUOX2 may be linked to the regulation of protease activity during inflammatory responses in UC. DUOX2 is positively correlated with ZC3H12A (r = 0.74). ZC3H12A is known for its role in the regulation of immune responses and inflammation [17]. The correlation suggests that DUOX2 and ZC3H12A may work together to modulate inflammatory pathways in UC (Table 6).

**Table 6:** Top Correlations with DUOX2. This table lists the top genes with the strongest correlations with DUOX2, indicating potential co-regulation or functional interactions in the context of UC.

Gene Symbol	Gene Title	Correlation (r)
LCN2	Lipocalin 2	0.86
MMP7	Matrix Metallopeptidase 7	0.82
PDZK1IP1	PDZK1 Interacting Protein 1	0.82
PI3	Peptidase Inhibitor 3	0.8
ZC3H12A	Zinc Finger CCCH-Type Containing 12A	0.74

### Pathway analysis

To further understand the biological processes and pathways associated with the differentially expressed genes, we performed Gene Ontology (GO) and KEGG pathway enrichment analyses. The results highlight several key pathways and biological processes that are significantly enriched in our dataset.

#### Interpretation of GO Pathway analysis results.

**Fig 5:**
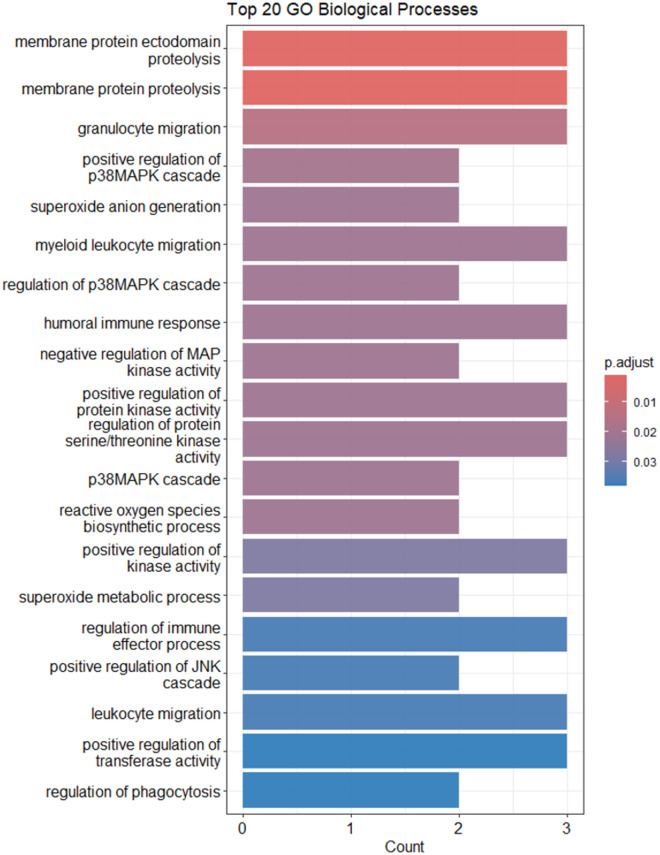
Enrichment analysis of gene expression data, top 20 KEGG pathways bar plot. This bar plot shows the top 20 enriched KEGG pathways. The x-axis represents the number of genes, while the y-axis lists the pathway names. The color of the bars reflects the adjusted p-value.

The pathway analysis results provide insights into the biological processes and molecular pathways that are significantly enriched in the differentially expressed genes associated with UC (Table 7) (Fig 5). Here, we focus on pathways that are relevant to DUOX2, a key gene identified in the analysis.

**Table 7:** Gene Ontology (GO) enrichment analysis. Table 1 shows the top GO terms enriched in the differentially expressed genes.

ID	Description	p-value	p.adjust	q-value	Gene names
GO:0006509	membrane protein ectodomain proteolysis	1.93 × 10^−6^	0.001	0.001	MMP7, IL1B, BACE2
GO:0033619	membrane protein proteolysis	4.71 × 10^−6^	0.002	0.001	MMP7, IL1B, BACE2
GO:1903596	regulation of gap junction assembly	1.39 × 10^−5^	0.003	0.002	CNTNAP2, IL1B
GO:0016264	gap junction assembly	4.70 × 10^−5^	0.008	0.004	CNTNAP2, IL1B
GO:0097530	granulocyte migration	8.71 × 10^−5^	0.012	0.006	CXCL2, IL1B, CD300A
GO:1900745	positive regulation of p38MAPK cascade	1.42 × 10^−4^	0.017	0.008	IL1B, NCF1
GO:0042554	superoxide anion generation	2.50 × 10^−4^	0.024	0.012	DUOX2, NCF1
GO:1900744	regulation of p38MAPK cascade	3.02 × 10^−4^	0.024	0.012	IL1B, NCF1
GO:0097529	myeloid leukocyte migration	3.19 × 10^−4^	0.024	0.012	CXCL2, IL1B, CD300A
GO:0006959	humoral immune response	3.84 × 10^−4^	0.024	0.012	PI3, CXCL2, IL1B
GO:0043407	negative regulation of MAP kinase activity	3.88 × 10^−4^	0.024	0.012	IL1B, CD300A
GO:0038066	p38MAPK cascade	4.85 × 10^−4^	0.024	0.012	IL1B, NCF1
GO:0045860	positive regulation of protein kinase activity	4.99 × 10^−4^	0.024	0.012	IL1B, NCF1, CD300A
GO:1903409	reactive oxygen species biosynthetic process	5.02 × 10^−4^	0.024	0.012	DUOX2, NCF1
GO:0071900	regulation of protein serine/threonine kinase activity	5.14 × 10^−4^	0.024	0.012	CCNO, IL1B, CD300A
GO:0006801	superoxide metabolic process	7.72 × 10^−4^	0.034	0.017	DUOX2, NCF1
GO:0033674	positive regulation of kinase activity	8.37 × 10^−4^	0.034	0.017	IL1B, NCF1, CD300A
GO:0046330	positive regulation of JNK cascade	1.15 × 10^−3^	0.045	0.023	IL1B, NCF1
GO:0002697	regulation of immune effector process	1.27 × 10^−3^	0.046	0.023	IL1B, NCF1, CD300A
GO:0050900	leukocyte migration	1.34 × 10^−3^	0.046	0.023	CXCL2, IL1B, CD300A
GO:0050764	regulation of phagocytosis	1.48 × 10^−3^	0.046	0.023	IL1B, CD300A

#### Key GO Pathways related to DUOX2.

Proteolysis (GO:0033619). DUOX2 is involved in generating reactive oxygen species (ROS) at the membrane level, which can lead to oxidative stress and protein modifications [18]. The enrichment of these pathways suggests that DUOX2 might be contributing to the proteolytic processes occurring at the cell membrane, which are crucial in signaling and immune responses in UC.

Granulocyte migration (GO:0097530). Granulocytes, such as neutrophils, play a vital role in the inflammatory response in UC [19]. DUOX2’s production of ROS can act as signaling molecules that promote the recruitment and activation of neutrophils [20]. This pathway’s enrichment highlights DUOX2’s potential role in modulating immune cell migration and inflammation in UC.

Chemokine Signaling Pathway (hsa04062). Chemokines are critical for immune cell trafficking [21]. DUOX2-generated ROS can enhance chemokine signaling, thereby influencing the migration and activation of immune cells [22]. This pathway’s enrichment underscores the importance of DUOX2 in driving inflammatory responses through chemokine signaling.

NOD-like Receptor Signaling Pathway (hsa04621). NOD-like receptors (NLRs) are intracellular sensors that detect pathogens and stress signals, leading to the activation of inflammatory responses [23]. DUOX2 may enhance NLR signaling by producing ROS [22], which can act as secondary messengers in these pathways. The enrichment of this pathway suggests a role for DUOX2 in innate immune responses in UC.

#### Implications for ulcerative colitis.

The enrichment of these pathways in the context of DUOX2 highlights its multifaceted role in the pathogenesis of UC.

Oxidative Stress and Inflammation: DUOX2-generated ROS contribute to oxidative stress, which can damage epithelial cells and exacerbate inflammation [24]. The proteolysis-related pathways suggest that DUOX2 might influence the degradation and modification of membrane proteins, impacting cell signaling and immune responses.

Immune Cell Recruitment and Activation: DUOX2 appears to play a crucial role in recruiting and activating immune cells, such as granulocytes, through chemokine signaling and other immune pathways [25,21]. This is particularly relevant in the context of UC, where uncontrolled immune responses lead to tissue damage and chronic inflammation.

## Results and discussion of pathway analysis

Our pathway analysis identified several significant pathways associated with the genes PLCB3, CXCL2, IL1B, and NCF1, which span multiple categories, including cardiovascular disease, infectious disease, immune system, endocrine system, sensory system, neurodegenerative disease, endocrine and metabolic disease, signal transduction, and development and regeneration.

**Fig 6:**
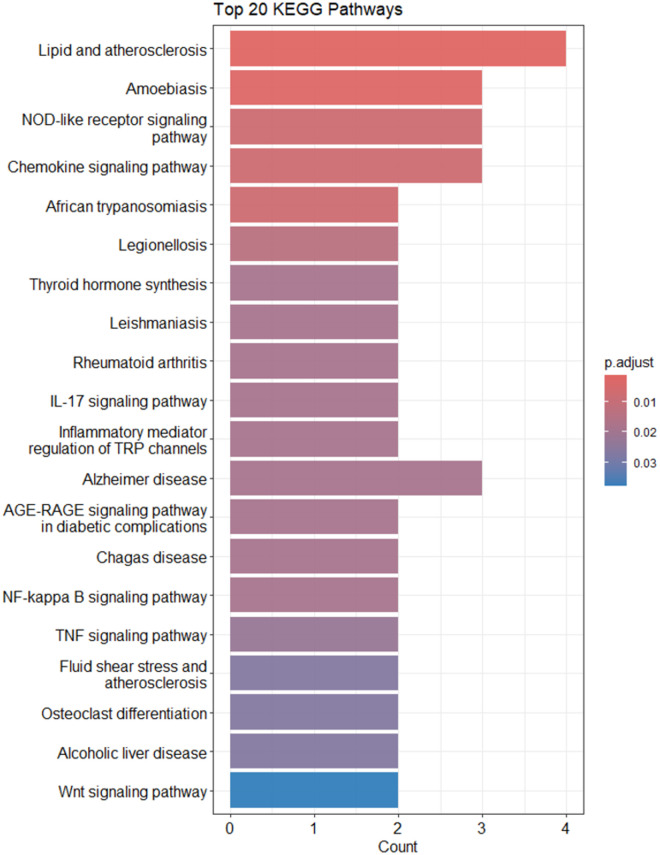
Enrichment analysis of gene expression data, top 20 GO biological processes bar plot. This bar plot highlights the top 20 enriched GO biological processes. The x-axis shows the number of genes, and the y-axis lists the biological process terms. The color of the bars corresponds to the adjusted p-value.

KEGG pathway enrichment analysis identifies significantly enriched pathways within a set of genes, helping to understand their biological functions. Our results, shown in Table 8 and Fig 6, revealed significant enrichment in the NOD-like receptor signaling pathway (p-value: 3.08E-04, adjusted p-value: 7.09E-03) and the NF-κB signaling pathway (p-value: 2.86E-03, adjusted p-value: 1.89E-02), with PLCB3 involved in both. These pathways are crucial for recognizing pathogens and initiating inflammatory responses, and their dysregulation is known to contribute to the pathogenesis of UC, where an inappropriate immune response to gut microbiota leads to chronic inflammation [26]. Additionally, the involvement of chemokine signaling pathways highlights the role of chemokines in recruiting immune cells to sites of inflammation [27]. Elevated levels of chemokines like CXCL2 can lead to excessive immune cell infiltration in the gut, exacerbating inflammation in UC [28].

**Table 8:** KEGG pathway enrichment analyses: Table 8 shows the top KEGG terms enriched in the differentially expressed genes.

Category	Description	pvalue	p.adjust	qvalue	Gene
Cardiovascular disease	Lipid and atherosclerosis	1.16 × 10^−5^	1.14 × 10^−3^	7.91 × 10^−4^	PLCB3, CXCL2, IL1B, NCF1
Infectious disease: parasitic	Amoebiasis	5.15 × 10^−5^	2.55 × 10^−3^	1.76 × 10^−3^	PLCB3, CXCL2, IL1B
Immune system	NOD-like receptor signaling pathway	3.08 × 10^−4^	7.09 × 10^−3^	4.90 × 10^−3^	PLCB3, CXCL2, IL1B
Immune system	Chemokine signaling pathway	3.38 × 10^−4^	7.09 × 10^−3^	4.90 × 10^−3^	PLCB3, CXCL2, NCF1
Infectious disease: parasitic	African trypanosomiasis	3.58 × 10^−4^	7.09 × 10^−3^	4.90 × 10^−3^	PLCB3, IL1B
Infectious disease: bacterial	Legionellosis	8.22 × 10^−4^	1.36 × 10^−2^	9.38 × 10^−3^	CXCL2, IL1B
Endocrine system	Thyroid hormone synthesis	1.47 × 10^−3^	1.89 × 10^−2^	1.31 × 10^−2^	PLCB3, DUOX2
Infectious disease: parasitic	Leishmaniasis	1.55 × 10^−3^	1.89 × 10 ⁻ ^−2^	1.31 × 10^−2^	IL1B, NCF1
Immune disease	Rheumatoid arthritis	2.25 × 10^−3^	1.89 × 10^−2^	1.31 × 10^−2^	CXCL2, IL1B
Immune system	IL-17 signaling pathway	2.30 × 10^−3^	1.89 × 10 ⁻^−2^	1.31 × 10^−2^	CXCL2, IL1B
Sensory system	Inflammatory mediator regulation of TRP channels	2.50 × 10^−3^	1.89 × 10^−2^	1.31 × 10^−2^	PLCB3, IL1B
Neurodegenerative disease	Alzheimer disease	2.55 × 10^−3^	1.89 × 10^−2^	1.31 × 10^−2^	PLCB3, IL1B, BACE2
Endocrine and metabolic disease	AGE-RAGE signaling pathway in diabetic complications	2.60 × 10^−3^	1.89 × 10^−2^	1.31 × 10^−2^	PLCB3, IL1B
Infectious disease: parasitic	Chagas disease	2.70 × 10^−3^	1.89 × 10^−2^	1.31 × 10^−2^	PLCB3, IL1B
Signal transduction	NF-κB signaling pathway	2.86 × 10^−3^	1.89 × 10^−2^	1.31 × 10^−2^	CXCL2, IL1B
Signal transduction	TNF signaling pathway	3.60 × 10^−3^	2.23 × 10^−2^	1.54 × 10^−2^	CXCL2, IL1B
Cardiovascular disease	Fluid shear stress and atherosclerosis	4.96 × 10^−3^	2.70 × 10^−2^	1.86 × 10^−2^	IL1B, NCF1
Development and regeneration	Osteoclast differentiation	5.10 × 10^−3^	2.70 × 10^−2^	1.86 × 10^−2^	IL1B, NCF1
Endocrine and metabolic disease	Alcoholic liver disease	5.17 × 10^−3^	2.70 × 10^−2^	1.86 × 10^−2^	CXCL2, IL1B
Signal transduction	Wnt signaling pathway	7.68 × 10^−3^	3.80 × 10^−2^	2.63 × 10^−2^	PLCB3, MMP7
Immune system	Neutrophil extracellular trap formation	9.20 × 10^−3^	4.34 × 10^−2^	3.00 × 10^−2^	PLCB3, NCF1
Cardiovascular disease	Diabetic cardiomyopathy	1.04 × 10^−2^	4.66 × 10^−2^	3.22 × 10^−2^	PLCB3, NCF1

The IL-17 signaling pathway also emerged as significant (p-value: 2.30E-03, adjusted p-value: 1.89E-02), further underscoring the pro-inflammatory environment characteristic of UC. IL-17 is crucial for promoting inflammation and has been associated with several autoimmune diseases, including UC [29]. Similarly, the TNF signaling pathway was found to be enriched, reflecting the critical role of TNF-alpha in UC [30]. This cytokine is a major inflammatory mediator, and anti-TNF therapies are commonly used in treating UC [31], highlighting the pathway’s relevance.

Furthermore, pathways related to lipid and atherosclerosis and fluid shear stress and atherosclerosis suggest a link between dysregulated lipid metabolism and inflammation, a hallmark of UC [32]. The involvement of pro-inflammatory cytokines like IL1B in these pathways suggests a connection to the chronic inflammatory state in UC.

Another significant finding is the neutrophil extracellular trap (NET) formation pathway. Neutrophils and their extracellular traps play a role in trapping pathogens but can also contribute to tissue damage and inflammation in UC [33]. Additionally, the AGE-RAGE signaling pathway in diabetic complications [34] and inflammatory mediator regulation of TRP channels were identified [35], indicating a broader impact of inflammatory signaling in UC pathology.

These pathways collectively highlight the complex interplay of inflammatory and immune responses in UC. The central genes, PLCB3, CXCL2, IL1B, and NCF1, are involved in multiple pathways, emphasizing their potential as therapeutic targets. The chronic inflammation seen in UC results from these intricate interactions, suggesting that a multi-targeted approach may be beneficial in managing the disease. This comprehensive understanding of the pathways involved provides valuable insights into the molecular mechanisms underlying UC and identifies potential avenues for therapeutic intervention.

## Discussion

This study aimed to explore the differential expression of genes in UC without considering the inflammation status among samples and further examining the inflammatory context. Our analysis identified several key genes with significant changes in expression levels, among which PLCB3 and DUOX2 were of particular interest due to their significantly different expression levels between normal and UC group.

### PLCB3 (Phospholipase C Beta 3)

PLCB3 was found to be significantly downregulated in UC samples compared to normal samples, with a log2 fold change of −1.59. This downregulation suggests that PLCB3 may play a protective role in maintaining intestinal homeostasis. PLCB3 is expressed in intestinal epithelial cells and immune cells, including T lymphocytes and macrophages, where it regulates cytokine production and cell signaling. PLCB3 is involved in the phosphoinositide signaling pathway, which is critical for various cellular functions, including cell proliferation, differentiation, and apoptosis [36]. The decreased expression of PLCB3 in UC could impair these processes, contributing to the pathological state observed in UC.

PLCΒ3 plays a multifaceted regulatory role in both immune signaling and epithelial barrier integrity, which may help explain its downregulation in UC. It hydrolyzes phosphatidylinositol 4,5-bisphosphate (PIP2) into two critical second messengers: inositol trisphosphate (IP3) and diacylglycerol (DAG). IP3 mediates calcium release from the endoplasmic reticulum, while DAG activates protein kinase C (PKC). These two branches of PLCΒ3 signaling converge on the activation of transcription factors such as NF-κB and AP-1, which are central to inflammatory responses [37]. Importantly, this same signaling cascade regulates cytoskeletal rearrangements and tight junction formation through PKC-mediated phosphorylation of structural proteins [38].

Pathway analysis revealed that PLCB3 is associated with several key pathways implicated in UC. Notably, the involvement in lipid and atherosclerosis pathways, the NOD-like receptor signaling pathway, and the NF-κB signaling pathway suggests a broad impact on inflammatory and immune responses. These pathways are essential for recognizing pathogens and initiating inflammatory responses, and their dysregulation can lead to chronic inflammation and tissue damage, characteristic of UC [39–41]. Thus, PLCΒ3 links immune activation, calcium dynamics, and structural remodeling through a shared signaling axis. In immune cells such as T lymphocytes and macrophages, PLCΒ3 contributes to antigen receptor signaling and cytokine production [37], while in intestinal epithelial cells, it modulates cell-cell adhesion and epithelial permeability. Decreased PLCΒ3 expression may therefore impair calcium- and PKC-mediated control of epithelial junctions, leading to barrier dysfunction and microbial translocation—hallmarks of UC pathogenesis [42]. These interconnected roles underscore PLCΒ3’s critical position at the interface of inflammation and mucosal integrity.

### DUOX2 (Dual Oxidase 2)

DUOX2 was significantly upregulated in UC samples, with a log2 fold change of 1.22, indicating its involvement in the inflammatory response. DUOX2 is an enzyme that produces reactive oxygen species (ROS), which play dual roles in cellular signaling and antimicrobial defense. However, excessive ROS can lead to oxidative stress, contributing to tissue damage and inflammation [43].

DUOX2 plays a pivotal role in both mucosal defense and pathological inflammation through its enzymatic production of hydrogen peroxide (H2O2), a reactive oxygen species (ROS). While ROS have antimicrobial properties, they also act as signaling molecules that activate key inflammatory cascades including NF-κB, MAPK, and JAK/STAT pathways. These interrelated signaling networks amplify inflammatory responses and promote cytokine production, creating a feedback loop that further upregulates DUOX2, especially under IFN-γ and LPS stimulation [21,44]. DUOX2 is highly expressed in colonic epithelial cells, where its overactivation contributes to oxidative stress. This oxidative burden damages tight junction proteins and cytoskeletal components responsible for maintaining epithelial barrier integrity, including occludin, ZO-1, and actin filaments. As a result, DUOX2-driven ROS production leads to barrier disruption and increased intestinal permeability—hallmarks of UC pathophysiology [38,43]. Among its associated pathways, chronic NF-κB activation appears particularly critical in UC progression, as it drives sustained inflammatory cytokine expression and tissue damage. Therefore, DUOX2 acts as both a sensor and amplifier of intestinal inflammation, linking innate immunity with structural degradation in the diseased mucosa.

The enrichment of pathways such as membrane protein ectodomain proteolysis and lipid and atherosclerosis in UC samples underscores key aspects of disease pathology. The former involves proteolytic cleavage of extracellular domains by enzymes like MMP7 and BACE2, which are known to mediate epithelial remodeling and immune cell recruitment [11,14]. Their upregulation suggests active extracellular matrix remodeling and inflammatory amplification in UC. The lipid and atherosclerosis pathway highlights immune-metabolic interactions involving lipid mediators, cytokines, and signaling enzymes such as PLCΒ3, suggesting a broader dysregulation of inflammatory homeostasis [32,45]. Although this pathway is classically linked to cardiovascular disease, it shares critical inflammatory mediators and may reflect systemic immune activation in UC.

### Relation of identified GO processes with ulcerative colitis

This study identified several GO Processes, of which, membrane protein ectodomain proteolysis, membrane protein proteolysis, and lipid and atherosclerosis are important to consider. Membrane protein ectodomain proteolysis involves proteolytic cleavage of the extracellular domain (ectodomain) from transmembrane proteins in a process called shedding. This modification controls the function and abundance of many membrane proteins, and affects cellular signaling, adhesion, and communication [46,47]. Membrane protein proteolysis includes the entire spectrum of proteolytic events involving membrane proteins. This includes both ectodomain shedding and other proteolytic cleavages, such as intramembrane proteolysis, which occurs within the membrane domain or at membrane surfaces and can lead to protein activation, inactivation, or degradation [48,49]. Lipid and atherosclerosis refer to atherosclerosis, which is a disease that is characterized by lipid accumulation and chronic inflammation in the arteries. Lipid signaling drivea plaque formation, vascular inflammation, foam cell formation, and the progression of cardiovascular disease [50–52]. These processes have important interactions. Membrane protein ectodomain proteolysis modifies cell communication and inflammation. By shedding these extracellular protein domains, such as receptors and adhesion molecules, membrane protein ectodomain proteolysis regulates inflammation and lipid uptake [46,49–51,53]. Membrane protein proteolysis modifies protein activity and abundance in both through ectodomain and intramembrane proteolysis which will affect key cell functions such as atherosclerosis which will be driven by lipid accumulation and inflammation [52].

The GO Processes membrane protein ectodomain proteolysis, membrane protein proteolysis, and lipid and atherosclerosis also have implications for ulcerative colitis. In ulcerative colitis, total cholesterol, HDL-C, LDL-C, and non-HDL-C, which are blood lipids are decreased in UC patients compared to healthy controls and correlate with ulcerative colitis severity [54–56].

These changes are correlated with active inflammation in the gut, disturbed metabolic balance, and potentially impaired lipid absorption. The altered lipid metabolism has been shown to affect immune signaling through PPARγ pathways, oxidative stress, and barrier function. All of these are important in ulcerative colitis pathology [54,57], Ulcerative colitis patients have been observed to have an increased risk of atherosclerosis and cardiovascular disease, most probably due to chronic systemic inflammation instead of hyperlipidemia [58].

### Limitations

While our findings identify strong transcriptional and pathway-level associations, we acknowledge the limitation of lacking direct in vivo or experimental validation. Functional assays such as knockdown or overexpression of PLCΒ3 and DUOX2 in colonic epithelial cells or murine models of colitis would be critical next steps to determine causality. These experiments could validate the role of these genes in barrier integrity, cytokine production, and disease progression. Additionally, applying CRISPR or siRNA approaches in organoid or enteroid models derived from UC patients could help verify the contribution of specific pathways (e.g., ROS production, tight junction remodeling) under controlled inflammatory conditions.

## Conclusions

In this study, we performed a comprehensive differential expression analysis to identify key genes involved in UC and their roles in the disease’s pathogenesis. The analysis indicated significant changes in gene expression between normal and UC samples, and highlighted PLCB3 and DUOX2 due to their distinct expression patterns and involvement in critical biological pathways.

PLCB3 was significantly downregulated in UC samples, which suggested its protective role in maintaining intestinal homeostasis. As part of the phosphoinositide signaling pathway, PLCB3 is crucial for various cellular functions, including proliferation, differentiation, and apoptosis [36]. Its decreased expression in UC likely impairs these processes and contributes to the disease’s pathology. Pathway analysis indicated that PLCB3 is associated with lipid and atherosclerosis pathways, the NOD-like receptor signaling pathway, and the NF-κB signaling pathway, all of which are essential for recognizing pathogens and initiating inflammatory responses [39]. The downregulation of PLCB3 could lead to dysregulation of these pathways, and result in chronic inflammation and tissue damage characteristic of UC. Furthermore, PLCB3 influences inflammatory pathways, immune cell function, epithelial barrier integrity, oxidative stress, and apoptosis, and emphasizes its multifaceted role in UC pathogenesis.

On the other hand, DUOX2 was significantly upregulated in UC samples, indicating its involvement in the inflammatory response. DUOX2, an enzyme that produces reactive oxygen species (ROS), plays dual roles in cellular signaling and antimicrobial defense, but excessive ROS can cause oxidative stress and tissue damage, exacerbating inflammation in UC [43]. Correlation analysis revealed strong positive associations between DUOX2 and several other genes, such as LCN2, MMP7, PDZK1IP1, PI3, and ZC3H12A, which suggested co-regulation and functional interactions within the inflammatory network of UC. DUOX2 is involved in pathways related to ROS production, which are significant in the context of oxidative stress and inflammation in UC [18].

The interplay between ROS generated by DUOX2 and the signaling pathways modulated by PLCB3could significantly influence inflammatory processes in UC. ROS can activate various signaling cascades, including those involving NF-κB, which is a key regulator of inflammatory responses [59]. PLCB3’s role in modulating calcium signaling and protein kinase C (PKC) activation might intersect with these pathways, thereby impacting the overall inflammatory milieu [60]. Understanding these interactions could provide deeper insights into their potential as therapeutic targets for UC.

Our pathway analysis identified several significant pathways associated with the differentially expressed genes, emphasizing their roles in inflammation, immune responses, and oxidative stress. The enrichment of pathways related to lipid metabolism, chemokine signaling, and immune cell recruitment highlights the complex interplay of these processes in UC pathogenesis. The central roles of PLCB3 and DUOX2 in these pathways suggest that targeting these enzymes and their downstream signaling pathways could offer therapeutic benefits in managing UC. Further research is necessary to fully elucidate their roles and develop specific interventions, with the ultimate goal of improving treatment strategies for UC patients.

## Supporting information

S1 File Differential_expression_results.csv – Comma separated file with differential expression results between normal and ulcerative colitis groups.
(CSV)

S2 File Differential_expression_with_inflammation.csv – Comma separated file differential expression results for normal and ulcerative colitis without inflammation groups.
(CSV)
